# Royal jelly promotes DAF-16-mediated proteostasis to tolerate β-amyloid toxicity in *C. elegans* model of Alzheimer's disease

**DOI:** 10.18632/oncotarget.10857

**Published:** 2016-07-26

**Authors:** Xiaoxia Wang, Min Cao, Yuqing Dong

**Affiliations:** ^1^ Department of Biological Sciences, Clemson University, Clemson, SC, USA; ^2^ Institute for Engaged Aging, Clemson University, Clemson, SC, USA

**Keywords:** Alzheimer's disease, royal jelly, β-amyloid, DAF-16, proteostasis, Gerotarget

## Abstract

Numerous studies have demonstrated that dietary intervention may promote health and help prevent Alzheimer's disease (AD). We recently reported that bee products of royal jelly (RJ) and enzyme-treated royal jelly (eRJ) were potent to promote healthy aging in *C. elegans*. Here, we examined whether RJ/eRJ consumption may benefit to mitigate the AD symptom in the disease model of *C. elegans*. Our results showed that RJ/eRJ supplementation significantly delayed the body paralysis in AD worms, suggesting the β-amyloid (Aβ) toxicity attenuation effects of RJ/eRJ. Genetic analyses suggested that RJ/eRJ-mediated alleviation of Aβ toxicity in AD worms required DAF-16, rather than HSF-1 and SKN-1, in an insulin/IGF signaling dependent manner. Moreover, RJ/eRJ modulated the transactivity of DAF-16 and dramatically improved the protein solubility in aged worms. Given protein solubility is a hallmark of healthy proteostasis, our findings demonstrated that RJ/eRJ supplementation improved proteostasis, and this promotion depended on the transactivity of DAF-16. Collectively, the present study not only elucidated the possible anti-AD mechanism of RJ/eRJ, but also provided evidence from a practical point of view to shed light on the extensive correlation of proteostasis and the prevention of neurodegenerative disorders.

## INTRODUCTION

Alzheimer's disease (AD) is the most observed neurodegenerative disorder in aged populations. Given age is the greatest risk factor for AD development, the aging of the baby-boom generation will lead to the explosive increase in the number of people with AD in the United States. As neuropathological lesions gradually impair the ability of thinking, communication, and memory [1–4], it is a big challenge for medical professionals to take care of AD patients. The steep cost of caring for these patients will be another significant impact on the healthcare system. Therefore, finding means to prevent or delay the onset of AD is one of the most urgent commitments in aging research.

A growing body of evidence showed that environmental stresses and cellular lesions may cause protein misfolding, aggregation, and amyloid formation, which are associated with protein toxicity and various disorders [5, 6]. Thus, aggregation and accumulation of aberrant proteins are shared common features of many age-related neurodegenerative diseases, although detailed molecular mechanisms of their pathogenesis remain unclear [7, 8]. AD is considered a proteotoxicity caused disease, which manifests obvious β-amyloid (Aβ) aggregations and Tau proteins nucleation [9, 10]. It is known that eukaryotic cells equipped a sophisticated network (proteostasis network) to maintain healthy proteostasis by eliminating aberrant proteins and concomitant proteotoxicity [11]. However, aging will perturb this regulatory network and decrease the protective capacity of proteostasis [12, 13]. Thus, developing therapeutic means to promote proteostasis network is a promising research avenue for the treatment of AD and other neurodegenerative disorders.

Numerous studies have shown that active ingredients from functional foods potently promote healthy aging [14–18], implying that dietary intervention may be a feasible means to prevent or delay the onset of AD. Royal jelly (RJ) and enzyme-treated RJ (eRJ) are consumed as functional foods due to their beneficial effects, such as antimicrobial, anti-inflammation, immune-modulation, and anti-oxidation [19, 20]. RJ is secreted by worker bees and used for developing and maintaining the queen bees [21]. eRJ is produced by digestion of RJ with a hydrolytic enzyme to decrease the allergenic properties [22]. We recently reported that supplementation of both RJ and eRJ significantly promoted lifespan and stress resistance in *C. elegans* [18]. Given that certain nutraceuticals with prolongevity effects may promote proteostasis and attenuate proteotoxicity [17], it is intriguing to test whether RJ/eRJ is able to influence the proteostasis network to prevent or delay AD.

*C. elegans* that model age-related disorders of neurodegeneration offers tremendous opportunities to study the molecular mechanisms of these diseases and to determine the effectiveness of therapeutic strategies [23–27]. To date, studies from worm models demonstrated that reduction of insulin/IGF signaling pathway (IIS) protected organisms from proteotoxicity [28–34]. Remarkably, these findings were reported to be conserved in mammalian animals [35–37]. CL2006 is a transgenic *C. elegans* in which human Aβ_1-42_ is constitutively expressed in the muscle cells. The subsequent aggregation of Aβ polypeptides leads to progressive body paralysis [30, 38], providing a unique platform to study AD and proteotoxicity. Here, we supplemented CL2006 worms with RJ/eRJ and investigated whether RJ/eRJ may help alleviate Aβ toxicity. Our results showed that RJ/eRJ dramatically delayed the progression of Aβ toxicity-induced paralysis and significantly reduced Aβ species in AD worms. Further genetic analyses showed that this anti-AD effect required DAF-16, rather than HSF-1 and SKN-1, in an IIS dependent manner. In addition, we found that RJ/eRJ supplementation substantially improved the protein solubility in aged worms. Considering that protein solubility associates with healthy proteostasis, these results demonstrated that RJ/eRJ acts through IIS and DAF-16 to promote proteostasis and mitigate proteotoxicity. Taken together, our findings not only revealed the possible molecular mechanism of RJ/eRJ-mediated protection against Aβ toxicity, but also highlighted the regulation of DAF-16 on the proteostasis network from a practical point of view.

## RESULTS

### RJ/eRJ supplementation delays the progression of paralysis in AD worms

**Figure 1:**
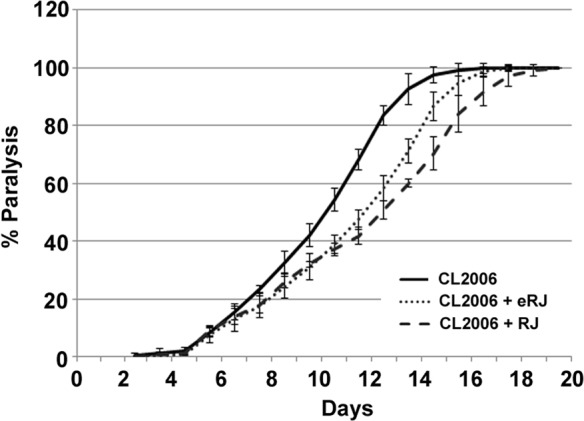
Supplementation of both RJ and eRJ mitigates Aβ toxicity in *C. elegans* The CL2006 worms treated with RJ (dashed line) and eRJ (dotted line) showed delayed progression of body paralysis as compared to the control worms (solid line). Each paralysis assay was conducted in triplicates and repeated at least three times with similar results. “% paralysis” indicates the average paralysis among the multi-replicates and error bars represent the standard deviation.

To determine whether RJ/eRJ possesses the beneficial effects to AD treatment, CL2006 worms were employed and the body paralysis was used as the readout to assess the degree of Aβ toxicity. As we previously reported that 2mg/ml RJ and 1mg/ml eRJ significantly prolonged *C. elegans* lifespan, the same concentrations of RJ and eRJ were adopted in this study [18]. In brief, synchronous CL2006 L4/young adult worms were treated with or without RJ/eRJ (2mg/mL and 1mg/mL, respectively) at 20°C and paralyses were measured every day until all AD worms were paralyzed. Our data indicated that RJ/eRJ supplementation dramatically delayed the progression of CL2006 worms' paralysis when compared with the non-supplemented controls (Figure 1). Considering that body paralysis is mainly triggered by Aβ toxicity, our finding suggested that RJ/eRJ could potently alleviate Aβ toxicity in AD worms.

### RJ/eRJ supplementation reduces the amount of Aβ species in AD worms

**Figure 2:**
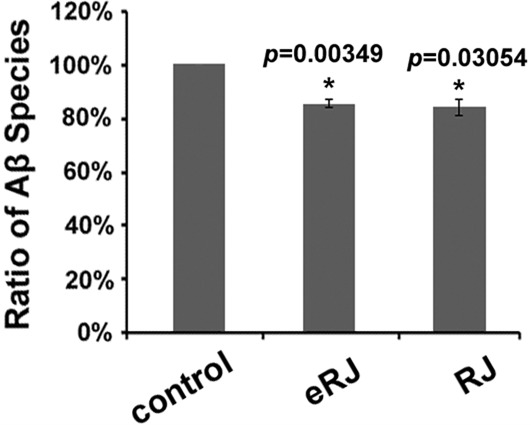
Supplementation of both RJ and eRJ reduces the amount of total Aβ species Western blot analysis was conducted using 10-day old CL2006 worms. Aβ species are quantified by using ImageJ software. A significant reduction of intensity is observed in the samples from worms that had been treated with RJ and eRJ. The graph shows the mean intensity of Aβ species and is the result of three independent experiments. *p* value was calculated using Student's *t*-test. *, *p* < 0.05 when compared to controls.

It is reported that many nutraceuticals delayed the development of AD by mitigating Aβ toxicity through decreasing Aβ species [17, 39–41]. We questioned whether RJ/eRJ was able to alleviate Aβ toxicity through a similar mechanism. To this end, synchronized CL2006 young adult worms were treated with or without RJ/eRJ (2mg/mL and 1mg/mL, respectively) for 10 days at 20°C. Afterward, Western blotting was performed to assay for the total amount of Aβ species in these 10-day aged worms. Data were analyzed using NIH ImageJ software, and our findings showed that the total amount Aβ species in AD worms treated with RJ and eRJ were 13.61% and 21.90% less than that in the control AD worms, which were statistically significant (*p* < 0.05, Figure 2). Given that the amount of Aβ species contribute to Aβ toxicity, our results suggested that RJ/eRJ attenuated Aβ toxicity, at least in partial, by reducing the level of Aβ species.

### IIS and DAF-16 are required in RJ/eRJ-mediated protection against Aβ toxicity

**Figure 3:**
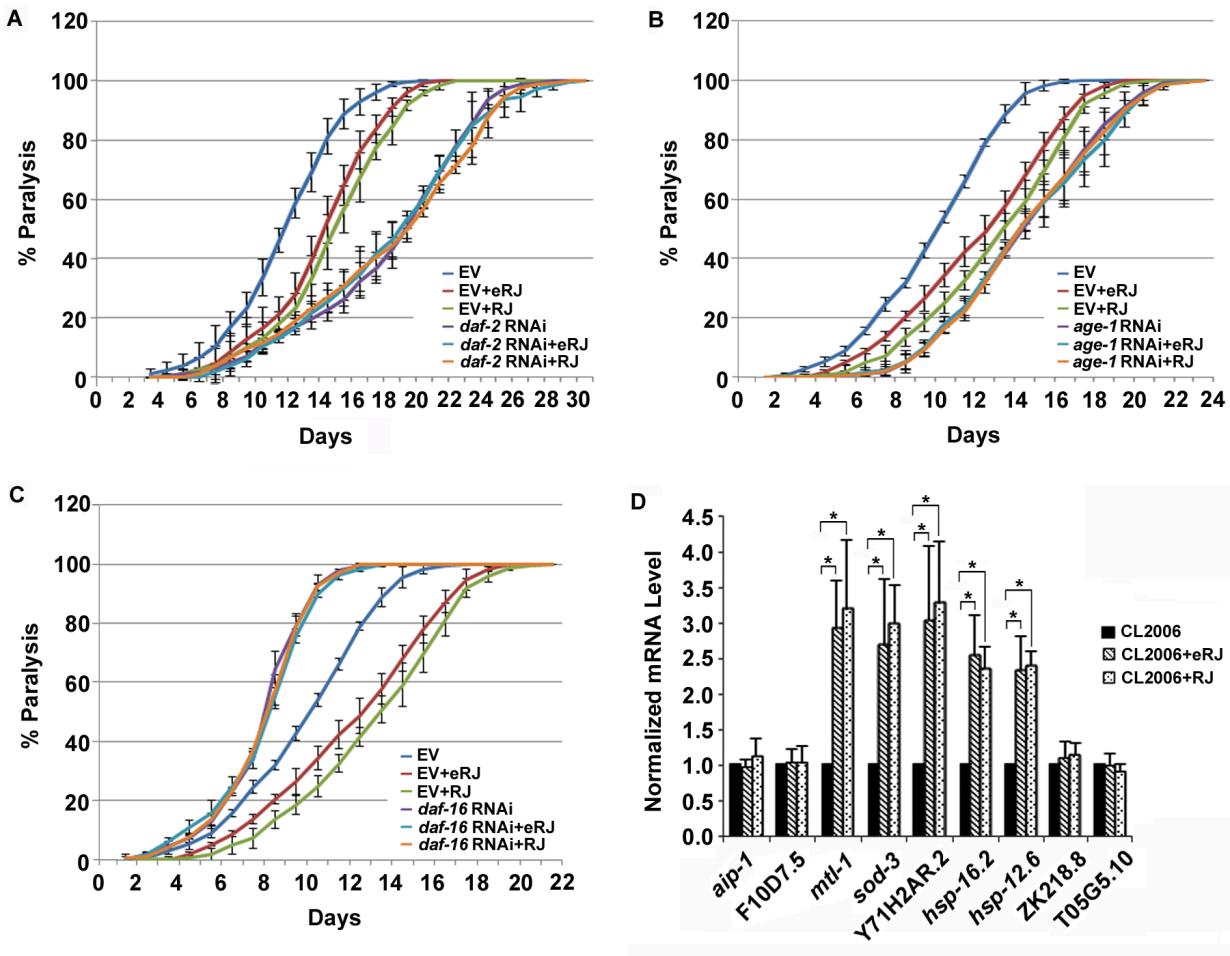
RJ/eRJ requires IIS and DAF-16 to protect against Aβ toxicity in CL2006 worms **A.** RJ/eRJ supplementation cannot further postpone the progression of body paralysis in CL2006 worms with reduced IIS by daf-2 RNAi. **B.** RJ/eRJ supplementation cannot further delay the progression of paralysis in CL2006 worms with reduced IIS by age-1 RNAi. **C.** Reducing DAF-16 by daf-16 RNAi significantly abolished the beneficial effects of RJ/eRJ delaying the progression of body paralysis in AD worms. Each paralysis assay was repeated at least three independent times with similar results. “% paralysis” indicates the average paralysis among the multi-replicates and error bars represent the standard deviation. **D.** The transcript levels of aip-1, F10D7.5, mtl-1, sod-3, Y71H2AR.2, ZK218.8, hsp-16.2, hsp-12.6, and T05G5.10 were measured using qRT-PCR in CL2006 worms supplemented with RJ/eRJ (2mg/ml and 1mg/ml, respectively). The data from three independent experiments were pooled to calculate the mean RNA level normalized to the internal control act-1. The standard errors of the mean (SEM) are shown. *, *p* < 0.05 when compared to non-treated controls.

It was reported that the reduction of IIS prevented neurodegeneration in mammals and worms by modulating the transactivities of its downstream transcription factors [42]. Our previous studies showed that IIS and DAF-16 played an essential role in RJ/eRJ-mediated lifespan extension and stress resistance [18]. Considering the potential link between prolongevity and anti-AD function, we wondered whether IIS and DAF-16 also played an imperative role in RJ/eRJ-mediated protection against Aβ toxicity. To address this, the paralysis progression of CL2006 worms supplemented with RJ/eRJ was monitored when the gene expression of *daf-2*, *age-1*, and *daf-16* were individually knocked down *via* RNA interference (RNAi). In detail, synchronous AD worms were collected from RJ/eRJ containing NGM plates seeded with particular aforementioned RNAi or a paired control (empty vector, EV) bacteria. When worms developed into L4/young adult, paralysis assays were performed every day until all AD worms were paralyzed. In our assays, RJ/eRJ supplementation still dramatically delayed the progression of paralysis in CL2006 worms treated with paired control RNAi. In contrast, RNAi of *daf-2*, *age-1*, and *daf-16* in CL2006 worms completely abolished RJ/eRJ-mediated beneficial effects on delaying the progression of paralysis (Figure 3A, 3B, and 3C). These findings together suggested that IIS and DAF-16 were essential in RJ/eRJ-mediated protective effect against Aβ toxicity.

As DAF-16 is a versatile transcription factor, it is very likely that the RJ/eRJ-mediated protection against Aβ toxicity may depend on the transactivity of DAF-16. To examine this speculation, synchronized CL2006 L4/young adult worms were treated with or without RJ/eRJ for 6 days, and some representative target genes of DAF-16 were selected to measure their expression levels, individually. Our results showed that *sod-3*, *mtl-1*, *Y71H2AR.2*, *hsp-16.2*, and *hsp-12.6* were significantly upregulated in CL2006 worms treated with RJ/eRJ when compared with non-supplemented controls (Figure 3D). These results not only indicated that RJ/eRJ protected against Aβ toxicity through modulating the transactivities of DAF-16, but also further confirmed our genetic analysis that *daf-16* was required by RJ/eRJ to delay the progression of paralysis in AD worms.

### HSF-1 and SKN-1 are dispensable in RJ/eRJ-mediated protection against Aβ toxicity

**Figure 4:**
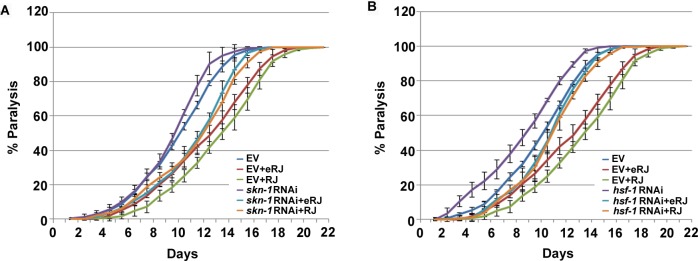
skn-1 and hsf-1 are dispensable in RJ/eRJ-mediated protection against Aβ toxicity **A.** RJ/eRJ supplementation can still delay the progression of Aβ toxicity-induced paralysis in AD worms with SKN-1level reduced by skn-1 RNAi. **B.** RJ/eRJ supplementation can still delay the progression of body paralysis in AD worms with HSF-1 level reduced by hsf-1 RNAi. Each paralysis assay was repeated at least three independent times with similar results. “% paralysis” indicates the average paralysis among the multi-replicates and error bars represent the standard deviation.

In addition to DAF-16, SKN-1 and HSF-1 are two other important transcription factors downstream of IIS, and also play an important role in alleviating Aβ toxicity in *C. elegans* [42]. Given that RJ/eRJ relied on IIS to alleviate Aβ toxicity, we were curious whether SKN-1 and HSF-1 may also contribute to this RJ/eRJ-mediated anti-AD effect, just like DAF-16. To this end, CL2006 worms supplemented with RJ/eRJ were fed with RNAi bacteria of *skn-1* and *hsf-1*, respectively. CL2006 worms fed with control RNAi (paired empty vector) bacteria served as controls. Intriguingly, our results showed that supplementation of RJ/eRJ still delayed the progression of paralysis in CL2006 worms fed with *skn-1* or *hsf-1* RNAi bacteria (Figure 4A and 4B). These results indicated that SKN-1 and HSF-1 might be dispensable in RJ/eRJ-mediated protection against Aβ toxicity.

### RJ/eRJ supplementation improved proteostasis in aged *C. elegans*

**Figure 5:**
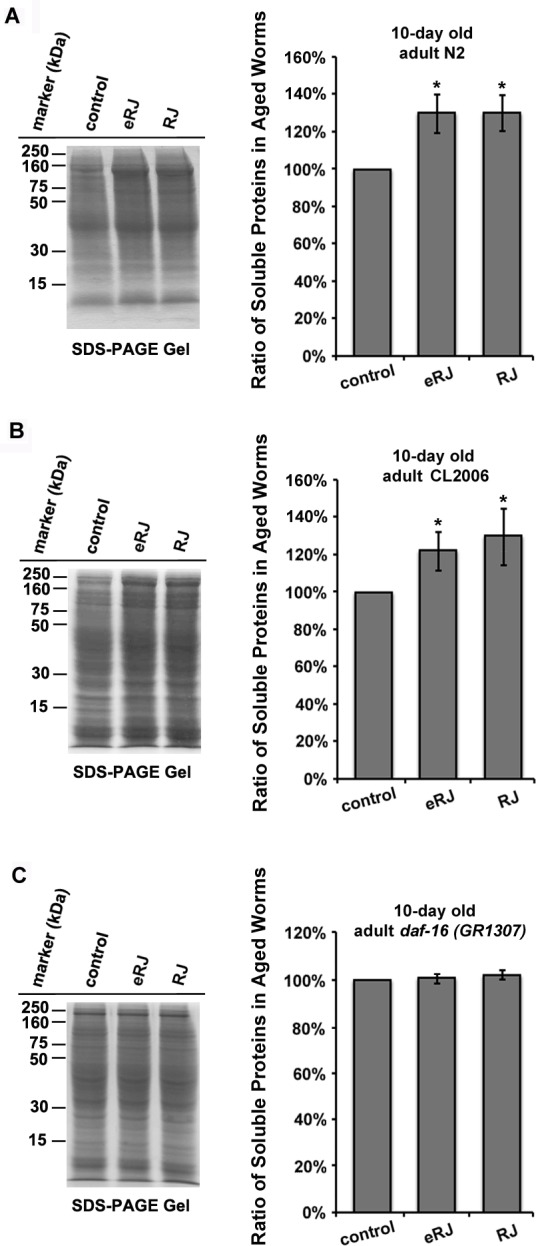
RJ/eRJ improves proteostasis in aged *C. elegans* in a DAF-16 dependent manner **A.** RJ/eRJ treatment increases protein solubility in 10-day old N2 worms. **B.** RJ/eRJ supplementation increases protein solubility in 10-day old CL2006 AD worms. **C.** RJ/eRJ treatment cannot increase protein solubility in 10-day old daf-16 (mgDf50) mutant worms. Soluble proteins on the SDS-PAGE gel are quantified by using ImageJ software. Data are expressed as mean intensity from three independent experiments. The standard errors of the mean (SEM) are shown. *p* value was calculated using Student's *t*-test. *, *p* < 0.05 when compared to controls.

Proteostasis machinery alleviates proteotoxicity to maintain the integrity of the proteome [43–47]. Given that RJ/eRJ consumption mitigated Aβ toxicity in AD worms, we speculated whether RJ/eRJ may modulate the proteostasis network to present anti-AD effect in aged populations. Thus, we tested the overall solubility of proteins in aged worms since protein solubility is a primary hallmark of healthy proteostasis [45, 48, 49]. Specifically, synchronized worms of wild type N2 and CL2006 treated with or without RJ/eRJ were harvested to extract total proteins. After sonication and high speed centrifugation, soluble fractions were isolated from the total proteins. SDS-PAGE and ImageJ were utilized to determine whether RJ/eRJ affected the solubility of proteins in aged *C. elegans*. Relative to non-supplemented controls, RJ/eRJ supplementation increased 26.95% (RJ) and 26.87% (eRJ) of soluble proteins in N2 worms (Figure 5A). Similarly, RJ/eRJ also displayed an average increase of 27.13% (RJ) and 21.27% (eRJ) of soluble proteins in CL2006 worms as compared to non-supplemented controls (Figure 5B). Together, these results suggested that RJ/eRJ profoundly improved proteostasis in aged *C. elegans*.

Given the fact that DAF-16 was essential in RJ/eRJ-mediated lifespan extension, stress resistance, and protection against Aβ toxicity, we speculated that DAF-16 may also participate in RJ/eRJ-mediated promotion of proteostasis and protein solubility. To test this speculation, synchronized *daf-16* (*mgDf50*) mutant worms were treated with or without RJ/eRJ for 10 days. Thereafter, worms were collected for total protein extraction. Sonication and centrifugation were utilized to remove the insoluble proteins, and the amount of soluble proteins was analyzed by SDS-PAGE and ImageJ software. Not surprisingly, RJ/eRJ-mediated improvement of protein solubility was abolished in the *daf-16* deletion mutant, suggesting the essential role of *daf-16* in RJ/eRJ-mediated promotion of proteostasis (Figure 5C).

## DISCUSSION

It has emerged that some anti-aging nutraceuticals could delay the development of neurodegenerative disorders. For instance, cranberry protected *C. elegans* from thermal stress and slowed down the development of AD [16, 50]. Antioxidant resveratrol not only had prolongevity effect but also delayed the development of Alzheimer's disease and Parkinson's disease [51, 52]. Our previous studies reported that RJ/eRJ supplementation not only extended *C. elegans* lifespan, but also increased their stress resistant capacity. This implied that RJ/eRJ consumption could lead to healthy aging in multicellular organisms. Hence, our present studies attempted to further investigate the potential beneficial effect of RJ/eRJ on AD treatment and its underlying molecular mechanisms. Strikingly, our results showed that RJ/eRJ supplementation delayed the progression of paralysis in AD worms. Further genetic analyses suggested that RJ/eRJ requires *daf-16*, rather than *skn-1* and *hsf-1*, to protect against Aβ toxicity. Mechanistic studies demonstrated that RJ/eRJ modulates the transactivities of DAF-16 through IIS cascade. Moreover, RJ/eRJ consumption resulted in the reduction of Aβ species in AD worms and greatly increased the solubility of proteins in aged worms [45, 48, 49]. Intriguingly, the improvement of protein solubility conferred by RJ/eRJ supplementation also required the function of DAF-16. Considering the correlation of protein solubility and proteostasis, and the imperative role of DAF-16 in regulating proteostasis, our findings suggested that RJ/eRJ may alleviate Aβ toxicity by promoting the function of proteostasis machinery in *C. elegans* through IIS and DAF-16.

It was reported that reduction of the IIS pathway prevents neurodegeneration in worms and mammals, and this preventive effect relies on the transactivities of three important transcription factors (*daf-16*, *skn-1*, and *hsf-1*) [42]. Given that IIS played an essential role in RJ/eRJ-mediated protection against Aβ toxicity (Figure 3), we thus wondered whether RJ/eRJ may preferentially influence the function of these downstream transcription factors contributing to the reduction of proteotoxicity. To address this concern, RNA interference was utilized to respectively knock down the gene expression of *daf-16*, *hsf-1*, and *skn-1*, respectively. Our genetic epistasis results showed that RNAi of either *skn-1* or *hsf-1* did not abolish RJ/eRJ-mediated progression delay of body paralysis (Figure 4A and 4B), while RNAi of *daf-16* in CL2006 AD worms completely abolished the progression delay of paralysis, relative to controls (Figure 3D). These findings indicated that RJ/eRJ's protection against Aβ toxicity in *C. elegans* is mainly dependent on DAF-16. Given that inefficiency of proteostasis machinery results in the accumulation of aggregates [43], such as Aβ aggregates, we reasoned that RJ/eRJ supplementation protected AD worms against Aβ toxicity by improving the regulation of proteostasis. Considering that DAF-16 plays a pivotal role in proteostasis network [28], our results suggested that RJ/eRJ supplementation selectively modulate the transactivity of DAF-16 to improve proteostasis in AD worms.

Ben-Zvi *et al*. demonstrated that collapse of proteostasis leads to impaired protein solubility and increased cytotoxicity [48]. Reis-Rodrigues and colleagues further claimed that accumulation of insoluble proteins with diverse biological functions may be a general feature of normal aging and late-onset neurodegenerative diseases [49]. Collectively, previous findings highlighted that a healthy proteostasis is associated with good protein solubility and attenuated proteotoxicity. Interestingly, our results showed that RJ/eRJ significantly increased protein solubility in both aged wild type worms and aged AD worms, which suggested that RJ/eRJ possessed the capability to help maintain a healthy proteostasis in *C. elegans*. Given that RJ/eRJ requires DAF-16 to protect against Aβ toxicity, we speculated that DAF-16 is also essential in RJ/eRJ-mediated healthy proteostasis maintenance. In support of our speculation, our further protein solubility assay showed that RJ/eRJ supplementation barely altered the protein solubility in aged *daf-16* deletion mutants. Taken together, our results attributed RJ/eRJ-mediated reduction of Aβ toxicity to DAF-16 and the improved proteostasis.

Given that most of, if not all, cellular functions depend on gene expression, the dependence of DAF-16 in RJ/eRJ-mediated proteostasis improvement proposed that RJ/eRJ may modulate the target gene expression of DAF-16 to influence proteostasis network. To address this, we selected several DAF-16 target genes and performed qRT-PCR to measure their expression in AD worms treated with or without RJ/eRJ. Intriguingly, RJ/eRJ supplementation increased the expression levels of *sod-3, mtl-1*, *hsp-12.6*, *hsp-16.2*, and *Y71H2AR.2* (Figure 3D). *sod-3* and *mtl-1* gene products play an important role in detoxification and homeostasis of ROS and heavy metal, respectively [53, 54]. *hsp-12.6* and *hsp-16.2* encode two essential chaperone proteins to prevent the aggregation of misfolded and unfolded proteins in response to proteotoxic stressors [55, 56]. Impressively, overexpression of HSP-16.2 could suppress the Aβ toxicity in AD worms. *Y71H2AR.2* encodes a protease, which may facilitate protein degradation through proteolysis [57]. Considering that stress response, molecular chaperone, and regulation of protein degradation are major components of the proteostasis network [11], our results implicated that RJ/eRJ may alleviate Aβ toxicity by modulating proteostasis network through DFA-16. It is also worth noticing that expression of other selected genes, such as *ZK218.8*, *T05G5.10*, *aip-1*, and *F10D7.5*, was barely influenced by RJ/eRJ supplementation (Figure 3D), although they are probably related to proteostasis network. *ZK218.8* and *T05G5.10* encode translation initiation factors and function in protein synthesis [57, 58], while *aip-1* and *F10D7.5* gene products function to facilitate toxic protein degradation under stress [59]. It was reported that RJ/eRJ-mediated prolongevity relied on fine-tune of DAF-16 transactivity through interplay with multiple proteins [18]. The expression preference of DAF-16 targets conferred by RJ/eRJ in AD worms actually suggested that RJ/eRJ may also fine-tune the transactivity of DAF-16, and in turn specifically up-regulate the expression of certain proteins associated with proteostasis network. It will be of great interest to decipher the RJ/eRJ-mediated fine-tune regulation on DAF-16 in AD worms. Of course, more experiments are needed to this end.

A growing body of evidence suggests that bioactive substances in natural foods interplay with cellular proteins, nuclear acids, and lipids by the chemical modification or physical interaction, to influence human health [60]. It is conceivable that the bioactive ingredients in RJ may function through the similar modes of action to ameliorate Aβ toxicity in animals. Given that RJ is a unique mixture rich of proteins, carbohydrates, lipids, minerals, and vitamins [61], in order to boost the therapeutic effects of RJ, it is critical to determine its functional components corresponding to the reduction of proteotoxicity in cells. Indeed, our finding that RJ/eRJ depends on DAF-16 to improve the function of proteostasis provides a simple and clear readout to determine these active components from RJ/eRJ.

Overall, our finding revealed the molecular mechanisms of RJ/eRJ-mediated alleviation of Aβ toxicity in *C. elegans*, and explicitly underscored the correlation of proteostasis maintenance and proteotoxicity reduction in cells. As both IIS/DAF-16(FOXOs) and proteostasis network are highly conserved in species ranging from *C. elegans* to human, the potential function of RJ/eRJ in AD treatment might also be beneficial to humans.

## MATERIALS AND METHODS

### Strains and growth conditions

All strains were maintained at 16°C on nematode growth medium (NGM) seeded with *Escherichia coli* OP50 feeding strain. Strains used in this study were as follows: N2 Bristol (wild type), CL2006 (AD worm), and *daf-16 (mgDf50)*. All the strains were obtained from the Caenorhabditis Genetics Center (CGC), University of Minnesota.

### Preparation of RJ and eRJ

The powder of RJ and eRJ were provided by Yamada Apiculture Center, Inc., Okayama, Japan. To prepare RJ and eRJ supplemented food, appropriate amount of RJ or eRJ in powder form were dissolved in sterile distilled water and suspended into the liquid NGM (2mg/mL RJ and 1mg/mL eRJ) one day before the assay [18].

### RNA interference

RNA interference (RNAi) clones were grown overnight at 37°C on Luria Broth plate in the presence of tetracycline (12.5 μg/ml) and carbenicillin (25 μg/ml). Bacterial colonies were inoculated and grown for 8-12 hours, then induced with 2mM isopropyl β-D-1-thiogalactopyranoside (IPTG) for 4 hours at 37°C. Ten-fold concentrated RNAi bacteria were seeded onto RNAi plates containing 25 μg/ml carbenicillin. The RNAi constructs targeting *daf-2, age-1, daf-16, hsf-1*, and *skn-1* were obtained from the *C. elegans* ORFeome RNAi library v1.1.

### Worm paralysis assays

The assays using strain CL2006 were carried out as described by Cohen and colleagues [28]. Synchronous populations of CL2006 worms were prepared on NGM plates by allowing 10-15 hermaphrodites lay eggs overnight at 16°C, and the parents were removed the next day. The eggs were allowed to hatch, and 30 L4/young adult worms per plate were used for each assay at 20°C. All paralysis plots were done in triplicates, and a minimum of three independent trials were performed per condition. Nematodes were scored as paralyzed if they exhibited “halos” of cleared bacteria around their heads (indicative of insufficient body movement to access food) or failed to undergo full body wave propagation upon the nose prodding. Worms were checked every day until all worms were paralyzed. The data were pooled, and the percentage of paralyzed worms was calculated and analyzed using Student's *t*-test. *p* < 0.05 was accepted as statistically significant.

### Western blotting of Aβ species

Rabbit polyclonal Aβ_1-42_ primary antibodies were from Abcam (ab39377). For Western blot analysis, CL2006 worms were synchronized by allowing 10-15 hermaphrodites to lay eggs overnight on OP50 seeded NGM plates at 16°C. The parents were removed, and eggs were allowed to hatch and develop to the L4 stage. Subsequently, worms were transferred to RJ (2mg/mL) / eRJ (1mg/mL) containing NGM plates and continued to grow at 20°C. CL2006 worms on regular NGM plates without RJ/eRJ served as control. After 10 days of growth, worms were transferred to micro-centrifuge tubes and washed with S-basal followed by protein immobilization on polyvinylidenefluoride (Bio-Rad) membrane. Polyvinylidenefluoride membrane was incubated with primary antibodies (1:1,000) diluted in 5% nonfat dry milk and then with secondary, HRP-conjugated goat anti-rabbit antibodies (Genscript, A00098; diluted 1:10,000). ACTIN was used as loading control, and the anti-ACTIN antibodies (MAB1501) were from EMD Millipore. Detection was undertaken with standard ECL protocol. Mean intensity of Aβ signals was analyzed using ImageJ software (National Institute of Health).

### Soluble protein extraction

The soluble protein extraction was performed as described previously with alterations [17, 49]. Synchronous populations of eggs were prepared by alkaline hypochlorite treatment of gravid adults grown at 16°C. Eggs were allowed to hatch and develop by transferring to OP50-seeded NGM plates containing RJ (2mg/mL) or eRJ (1mg/mL) at 20°C. Eggs hatched and developed on OP50-seeded NGM plates without RJ/eRJ served as controls. Both worms treated with or without RJ/eRJ were collected 10 days after L4 stage. Three separate replicates of each sample (about 200mg [wet weight] of worms) were collected. Total protein extracts were produced in phosphate-buffered saline by sonication on ice, and then the total protein concentration was determined by conducting a bicinchoninic acid assay. Next, the normalized protein samples were spun for 10 minutes at 14,000*g* to remove the insoluble fraction. The same volume of supernatants (soluble fraction) was loaded and analyzed by sodium dodecyl sulfate-polyacrylamide gel electrophoresis. Mean intensities of protein bands were analyzed using ImageJ software (National Institute of Health).

### Gene expression analysis by quantitative real-time PCR

CL2006 worms were synchronized by allowing 10-15 hermaphrodites to lay eggs for 6 hours on OP50 seeded NGM plates at 16°C. The eggs were allowed to hatch and develop. The L4 /young adult worms were transferred to RJ/eRJ (2 and 1mg/mL respectively) NGM plates (containing 50 μg/mL FUDR to prevent the growth of progeny) to maintain for 6 days. CL2006 worms on regular NGM (containing FUDR) plates without RJ/eRJ served as controls. Worms at the 6-day old stage were collected with M9 buffer into a 50-100-μl pellet. RNA was prepared using RNAzol^®^ RT reagent (Molecular Research Center) and stored at −80°C. Complementary DNA was prepared by using Invitrogen Superscript^TM^ first-strand synthesis system for RT-PCR (Invitrogen). Real-time PCR was performed using using SsoFast^TM^ EvaGreen^®^ Supermix (Bio-Rad) and the CFX96^TM^ real-time PCR detection system according to the manufacturer suggested protocol (Bio-Rad). The quantitative real-time PCR (qRT-PCR) conditions were as follows: 95°C for 3 minutes, followed by 40 cycles of 10 seconds at 95°C, and 30 seconds at 60°C. *act-1* was used as an internal control to normalize the expression level of target transcripts. Relative fold-changes for transcripts were calculated using the comparative *C*_T_(2^−ΔΔCT^) method (43). Each qRT-PCR experiment was repeated three times using independent RNA/cDNA preparations. The data were pooled and analyzed using student's *t*-test, and a *p* value < 0.05 was accepted as statistically significant. The qRT-PCR primers for *hsp-12.6* are as follows: 5′-ATGATGAGCGTTCCAGTGATGGCTGACG-3′ (F) and5′-TTAATGCATTTTTCTTGCTTCAATGTGAAGAATTCC-3′ (R). Primers for *hsp-16.2* are as follows: 5′-TTGCCATCAATCT CAACGTC-3′ (F) and 5′-CTTTCTTTGGCGCTTCAATC-3′ (R). Primers for *sod-3* are as follows: 5′-CCAACCAGCGCTG-AAATT CAATGG-3′ (F) and 5′-GGAACCGAAGTCGCGCTTAATAGT-3′ (R). Primers for *mtl-1* are as follows: 5′-ATGGCTTGCAAGTGTGACTG-3′ (F) and 5′-CACATTTGTCTCCGCACTTG-3′ (R). Primers for *aip-1* are as follows: 5′- GAGCGGGATCACAGTTGTGAG-3′ (F) and 5′- GATGTGATTGAATCCGTCCAG-3′ (R). Primers for *F10D7.5* are as follows: 5′- GCACTAGAGGACCATTACAGTT-3′ (F) and 5′- CGTTCTCGTCAATCTCGATAGG-3′ (R). Primers for *ZK218.8* are as follows: 5′- TGCTACTGGCTGTGTGTTAG-3′ (F) and 5′- CAATAGTTCCGGCATTCACATAAT-3′ (R). Primers for *T05G5.10* are as follows: 5′- GGAGACTTGGGAAACACTATCC-3′ (F) and 5′- TGTATCCGAGAATAGCCTCCT-3′ (R). Primers for *Y71H2AR.2* are as follows: 5′- GAGTTGTGGCAGAGGGTAATG-3′ (F) and 5′- GGATGGGTTGTAGATTCCGATTT-3′ (R). Primers for *act-1*are as follows: 5′- CCAGGAATTGCTGATCGTATGCAGAA-3′ (F) and 5′-TGGAGAGGGAAGCGAGGATAGA-3′ (R).
